# Research on the influence of perceived quality on users’ continuance usage intention of online live streaming class platforms: the mediating role of flow experience and the moderating impact of perceived usefulness

**DOI:** 10.3389/fpsyg.2024.1455597

**Published:** 2024-10-30

**Authors:** Fan Wu, Guangying Xie

**Affiliations:** ^1^International College, Dhurakij Pundit University, Bangkok, Thailand; ^2^School of Economics and Management, China University of Mining and Technology, Xuzhou, China

**Keywords:** perceived quality, flow experience, continuance usage intention, perceived usefulness, online live streaming class platforms

## Abstract

**Background:**

With the penetration of the “Internet+” into social life, the digitization of education has become a trend and national demand. Alongside the rise of the “Everyone Live Streaming” era, online live streaming class platforms have rapidly grown due to favorable user experiences.

**Methods:**

Grounded in the Expectation Confirmation Theory, this study employed questionnaire surveys to investigate the influence mechanism of perceived quality, flow experience, and perceived usefulness on individuals’ continuance usage intention of online live streaming class platforms.

**Results:**

Through the analysis of 773 survey responses, this study reveals several key findings. Firstly, perceived quality significantly and positively affects users’ intention to continue using the online live streaming class platforms. Secondly, flow experience partially mediates the relationship between perceived quality and continuance usage intention, underscoring its significance in user decision-making. Additionally, Perceived usefulness negatively moderates the relationship between perceived quality and flow experience, demonstrating varied effects.

**Conclusion:**

On the one hand, online live streaming platforms should actively invest in improving the quality of user perception, as this can lead to a better user flow experience and continued willingness to use. On the other hand, they should also pay more attention to the user’s flow experience, which has a direct impact on the user’s propensity to continue to use. Besides, the perceived usefulness of the online live class by the user should also be taken seriously.

## 1 Introduction

With the widespread of the internet in China, web surfing has become a part of people’s daily lives. Different from traditional audio and video formats, webcast services can instantly transmit images and sounds through a variety of communication technologies, allowing users to interact with streaming media in real time and influence users’ behavioral intentions (Wongkitrungrueng et al., 2020). With the continuous penetration of mobile networks and personal smart terminals into public life, online live broadcasts, with their interactivity, quasi-authenticity, borderlessness, and interconnectedness, have significantly affected social media users’ usage patterns and information dissemination willingness. To a certain extent, there has been a new phenomenon of “no online live broadcast, no communication.” Online live streaming class platforms have played an important role in the development of “Internet + Education,” enabling connections between teachers and students and providing better information adaptation and deeper interaction than traditional video courses recorded in advance experience. These platforms are characterized by their main functions such as real-time interactivity, flexible learning environments, multimedia content, personalized experiences, efficient playback, intelligent management, and social learning capabilities. They cater to a user base with fragmented study times, preference for “light course” methods, significant demands for professional and hobby-related learning, and a medium to high willingness to pay. These platforms offer a diverse range of live course content, including language learning, vocational skills, and culture and arts, to satisfy a variety of learning needs. Furthermore, they aggregate high-quality educational resources, facilitate self-learning, employ live streaming as the primary mode of delivery with interactive and recorded sessions, and homework submissions, overcoming geographical and temporal barriers to education, reducing costs, and fostering the dissemination of quality educational content. However, online live courses have completely different time and space boundaries and learning environments from traditional classroom teaching. Problems that may exist in traditional classroom teaching, such as students’ low interest in learning, low participation, and low achievement of teaching goals, are even more prominent in current online teaching. Therefore, in the context of online live streaming classrooms, it is particularly important and difficult to improve users’ willingness to continue using (Yuk-Chiu, 2023; Yuan and Gao, 2023; Wang and Lin, 2023).

However, as a network platform, online live streaming classrooms’ rapid development largely depends on users’ repeated use and word-of-mouth communication (Babić Rosario et al., 2016; Moriuchi and Takahashi, 2017). In view of this, this article intends to explore the factors that affect the continued use behavior of online live streaming class platforms users based on the Information System Continuance of Expectation Confirmation Model (ECM-ISC) proposed by Bhattacherjee (2001). According to the ECM-ISC model, the formation of emotions may lead to dependence on the intention to continue using. Cognition, emotion, and behavioral intention are the key mechanisms followed by this model. In the ECM-ISC model, Bhattacherjee (2001) considers satisfaction to be a short-term emotion related to previous adoption experiences with a specific matter during the continuous adoption phase. However, in the context of this study, it is not sufficient to explain the situation in high involvement application scenarios. In addition, given that the expectation confirmation model only considers the impact of a few traditional factors such as users’ rational cognitive factors (expectation confirmation and perceived usefulness) and attitudinal factors (satisfaction) on the continuance usage intention of information systems, it ignores the subjective feeling factors of users such as users’ experience and social influence.

According to the original definition of ECM-ISC, confirmation of expectations refers to the user’s comparison of the utility they experience when using a product or receiving a service with their expectations, to assess the consistency between perceived utility and expected utility. It is evident that enhancing the perceived quality of the platform helps to improve perceived utility, thereby positively affecting confirmation of expectations. To further investigate the specific factors affecting users’ intention to continue using, this study opts to use perceived quality in place of confirmation of expectations and concretizes the factors affecting confirmation of expectations in the expectation model into variables affecting the perception of platform quality, to further confirm their impact on users’ intention to continue using. On the other hand, the flow theory, originally applicable to individual psychological research, has also been widely applied to the study of online activities, so as to study the flow experience of internet users and explain their online behavior (Yeh et al., 2019; Chen, 2006). These studies show that the generation of users’ flow experience helps to positively influence their continued use behavior. This paper aims to study the intention of users to continue using online live streaming class platforms, and we plan to introduce the concept of flow experience to replace satisfaction, to explain the emotional experience of users participating in platform courses in the online live streaming class scenario. Therefore, this article adds two factors that reflect user experience, namely perceived quality and flow experience, to the model to enhance the explanatory power of the ECM-ISC model, which is also the main theoretical contribution of this study.

This paper innovatively expands the ECM-ISC model by introducing the theory of perceived quality and flow experience to enhance the model’s explanatory power regarding user retention on live streaming class platforms. First, we concretize the confirmation of expectations in the ECM-ISC model as users’ perception of the platform’s product and service quality, thereby more accurately capturing users’ satisfaction and loyalty towards the platform. Second, the introduction of flow experience theory allows us to more comprehensively understand the decision-making process of users in high involvement situations, which includes not only rational factors but also emotional factors. Furthermore, the introduction of the moderating variable perceived usefulness supplements the boundary conditions of the ECM-ISC theory’s research on factors affecting users’ intention to continue using by emphasizing the impact of users’ long-term impressions on flow experience in the context of live streaming classes and making the model’s explanation of users’ intention to continue using live streaming class platforms more refined. Finally, this paper also provides theoretical guidance for online live streaming class platforms and similar online education operators to improve services and better retain users.

The rest of this paper is organized as follows: Section 2 conducts the literature review and the derivation of the theoretical model. Section 3 introduces the research method, questionnaire design and data collection. Section 4 shows the statistical results and discusses the findings. Section 5 summarizes these insights and concludes the paper with a discussion of research limitations and future directions.

## 2 Literature review and hypotheses development

The model of user’s intention to continue using the live streaming class platform in this study includes four potential variables of perceived quality, flow experience, perceived usefulness, and continuance usage intention. To ensure the authority and continuity of the research, each variable is defined with reference to relevant academic literature and combined with the special context of live streaming class platforms. In them, perceived quality is defined as the user’s subjective evaluation and estimation of the overall quality of the user’s participation in online courses and offline activities (Agarwal and Teas, 2002; Dodds et al., 1991). Flow experience is a kind of optimized experience, which refers to the state where a person is fully engaged in the activity they are engaged in, forgetting worries, and filled with pleasure. Even without tangible benefits, they tend to seek to repeat this pleasant experience (Yeh et al., 2019; Chen, 2006). In contrast, perceived usefulness refers to the extent to which users believe that live streaming classes can improve their job performance or quality of life. It is a core concept in the Technology Acceptance Model (TAM), which reflects the user’s subjective judgment of practicality (Yan et al., 2021; Gefen et al., 2003; Bhattacherjee, 2001). Continuance usage intention is a kind of psychological state of intention to continue using the platform that users formed after using the live streaming class platform for a period of time (You et al., 2020; Bhattacherjee, 2001). We recognize that while perceived quality is an important factor affecting users’ perception of product usefulness, it is not the only determining factor. Users may be inclined to consider it useful because of the platform’s high perceived quality, but this does not mean that high perceived quality always leads to high perceived usefulness. For example, even if a product is of high quality, if its functions do not match the user’s needs, then the user may not consider the product useful to them.

### 2.1 Perceived quality and continuance usage intention

Zeithaml (1988) argues that consumers’ perception of quality arises from intrinsic and extrinsic cues. Between them, intrinsic cues refer to the physical attributes of a product. For instance, in the case of juice products, these attributes include taste, color, texture, and sweetness. Extrinsic cues, on the other hand, are related to the product but not its inherent components. Factors such as price, brand name, and advertising level are commonly used by consumers to assess product quality (Zeithaml, 1988). Drawing on this definition of perceived quality and considering the characteristics of users in the context of online live streaming class platforms, this study defines perceived quality as users’ subjective evaluation and estimation of overall quality throughout their participation in online courses and offline activities. Perceived quality can be assessed across four dimensions: platform information quality, platform system quality, platform service quality, and course product and service quality (Bolton and Drew, 1991). The higher users evaluate the overall quality, the more value they perceive, thereby influencing their purchase intention. In terms of platform information quality, high-perceived quality in live streaming courses often entails superior teaching content and abundant learning resources. Users can acquire valuable knowledge in the courses, effectively achieving their learning objectives. High-quality content meets users’ needs and makes them more willing to continue using the platform (Salloum et al., 2019; Li and Shang, 2020). Besides, high-quality online courses also typically provide better opportunities for social interaction, such as online discussions and Q&A sessions. Users can engage with peers, instructors, and fellow learners, sharing experiences and perspectives. This social interaction enhances the learning experience and encourages users to continue using the platform (Kervenoael et al., 2020). In terms of platform system quality, high-perceived quality in live streaming courses often includes better audiovisual quality, smooth playback, and interactive features. Users can view and listen to course content more clearly and interact more seamlessly with presenters or other participants, enhancing the learning experience. Such positive experiences stimulate users’ interest in learning and increase their intention to continue using the platform (Kervenoael et al., 2020).

In terms of platform service quality, a live streaming class platform with high perceived quality can establish trust among users. Users may feel that they are participating in a reliable and trustworthy learning environment. Trust can encourage users to recommend the platform to others, thereby expanding the user base (Atulkar, 2020). High-perceived quality in live streaming courses often stimulates users’ engagement and enthusiasm. Users may become more actively involved in course activities, submitting assignments, participating in discussions, and more, thereby deepening their connection and involvement with the platform (Barari et al., 2021). Regarding course product and service quality, users’ perceived quality of live streaming courses directly impacts their satisfaction. Satisfied users are more likely to continue using the platform, as they wish to continue receiving similar learning experiences and value (Li and Shang, 2020). In conclusion, perceived quality has a significant positive impact on the continuance usage intention of users on live streaming class platforms. By providing high-quality interactive experiences, content, user satisfaction, trust, and social opportunities, online live streaming class platforms can ignite users’ interest, increase their engagement, and thereby encourage them to use the platform over the long term.

Therefore, we proposed the following hypothesis:

H1: Perceived quality positively impacts users’ continuance usage intention of online live streaming class platforms.

### 2.2 Perceived quality and flow experience

On one hand, flow is a psychological state of complete immersion and focused attention in a certain activity, typically leading to feelings of pleasure, satisfaction, and fulfillment (Csikszentmihalyi, 2014). On the other hand, perceived quality refers to users’ subjective evaluations of platform information quality, platform system quality, platform service quality, and product and service quality (Bolton and Drew, 1991). Relevant research in the field of information systems user retention indicates that users’ perceived quality of system usage positively influences flow experience (Li and Shang, 2020). Learners’ subjective evaluations of course content, teaching methods, and technological performance contribute to perceived quality (Bolton and Drew, 1991), directly affecting whether users can engage fully in system usage. When platform content is appealing and challenging, it may stimulate learners’ interest and motivation (Cuevas et al., 2021), encouraging deeper engagement and facilitating entry into a state of flow. Additionally, well-organized interactions such as questioning, discussions, and group activities may enhance user involvement, increase engagement, and contribute to achieving flow (Zhao et al., 2020). Moreover, stable and smooth technological performance, such as clear visuals and sound, creates a comfortable learning environment, allowing learners to focus on content and more easily enter a state of flow (Ming et al., 2021).

Personalized attention and positive feedback are also important factors influencing the flow experience (Xue et al., 2020). By catering to the needs and interests of different learners, courses can stimulate their learning interests and encourage deeper engagement. Teachers who promptly address learners’ questions during live courses and provide positive encouragement and recognition can boost learners’ confidence and sense of achievement, contributing to entering a state of flow (Hong et al., 2019). In summary, in live courses, creating engaging content, interactive teaching methods, a stable and smooth technical environment, as well as personalized attention and feedback, can actively enhance learners’ perceived quality. This, in turn, increases the likelihood of them entering a state of flow.

Therefore, we proposed the following hypothesis:

H2: Perceived quality positively impacts user’s flow experience.

### 2.3 Flow experience and continuance usage intention

In online teaching system researches, flow experience has been demonstrated to have a positive and significant impact on users’ continuance usage intention (Mulik et al., 2020). Flow is a psychological state where individuals are fully immersed and deeply engaged in an activity, leading to feelings of pleasure, smoothness, and satisfaction (Csikszentmihalyi, 2014). Many literatures indicate that flow experience may positively influences users’ continuance usage intention in the context of live streaming courses from cognitive, emotional, and behavioral perspectives.

Firstly, at the cognitive level, flow experience can enhance users’ perceived value and learning effectiveness of live streaming courses. When users are in a state of flow, they are more likely to wholeheartedly engage with the course content, leading to a deeper understanding and application of knowledge (Zhao et al., 2020). This highly immersive learning approach contributes to improved learning efficiency, enhancing learners’ knowledge accumulation and skill enhancement. Additionally, Flow experience enhances users’ perception of course quality through five aspects.

1. Increased Focus. Users in a state of flow can understand course content more deeply. This enhancement in focus allows them to absorb information more comprehensively.
2. Time Perception Distortion. During the flow experience, users might feel that time passes more quickly. This distortion in time perception can make them feel that the course is more fulfilling and valuable.
3. Enhanced Intrinsic Motivation. Flow experiences are typically associated with intrinsic motivation, where users learn because they enjoy the learning process itself. This enhancement in intrinsic motivation can increase their interest and satisfaction with the course content.
4. Sense of Achievement. Users in a flow experience often have a sense of achievement after completing tasks, which can be translated into a positive evaluation of course quality.
5. Skill Improvement. Users in a flow experience enhance their skills by challenging and overcoming difficulties. This improvement in skills can make them feel the effectiveness of the course. As users experience a sense of achievement in their learning, they also develop a stronger recognition of the quality and value of the course, thereby reinforcing their desire for continued usage.

Secondly, at the emotional level, flow experience can create feelings of pleasure and satisfaction (Wu et al., 2020; Gao and Bai, 2014; Kazancoglu and Demir, 2021). Users in a state of flow experience emotional enjoyment as they immerse themselves in the learning process, fostering a positive emotional connection. This pleasurable experience becomes associated with the live streaming course, forming a positive emotional attachment. Over time, users’ pursuit of this pleasurable experience becomes a driving force for their continued usage, as they seek to re-experience the joy and fulfillment brought about by the flow state (Kang et al., 2018; Kim and Hall, 2019; Kim et al., 2017; Liu et al., 2016; Zhou, 2020).

Thirdly, at the behavioral level, the flow experience can enhance users’ intention to continue using the platform. Sustained flow experiences can cultivate emotional involvement and loyalty among users (Arghashi and Yuksel, 2022). The positive emotional connection established by users in the flow state influences their decision-making, making them more inclined to engage in learning activities in the future. Moreover, users who continue to use the platform are more likely to repeatedly enter the flow state, creating a virtuous cycle that further enhances the motivation for continuance usage intention.

In summary, in the context of online live streaming class platforms, the flow experience exerts a significant positive influence on users’ sustained engagement by enhancing cognitive value, fostering positive emotions, and reinforcing the behavioral motivation for continuance usage intention. Educators and course designers can optimize live courses by focusing on elements that contribute to the flow experience, such as challenging tasks, personalized feedback, and the creation of enjoyable experiences. This optimization can further stimulate users’ ongoing learning and participation.

Therefore, we proposed the following hypothesis:

H3: Flow experience positively impacts users’ continuance usage intention of online live streaming class platforms.

Through the above analyses we may find that flow experience serves as a mediating variable, acting as a bridge between perceived quality and continuance usage intention. Specifically, when users have a high perceived quality of the platform or service, they are more likely to experience a state of flow during their interaction with the platform, which is a psychological state of complete immersion and enjoyment. The emergence of flow experience enhances users’ sense of immersion and satisfaction, thereby increasing their satisfaction and loyalty to the platform. This positive change in psychological experience, in turn, promotes the formation of users’ intention to continue using. That is, flow experience is not only directly influenced by perceived quality but also plays a mediating role in the process of users forming an intention to continue using.

Therefore, we propose the following hypothesis:

H4: Flow experience mediates the relationship between perceived quality and continuance usage intention.

### 2.4 Perceived usefulness’ s moderating effect between perceived quality and flow experience

Perceived usefulness is a state of complete immersion in an activity which usually accompanied by a high level of focus and enjoyment (Csikszentmihalyi, 1990). It plays an important role in the formation of flow experience and the influence paths from perceived value to flow experience (Yang and Lee, 2023). In the prerequisite or preparatory stage of flow, Perceived usefulness is a key factor in users’ decisions on whether to invest time and effort. If users believe that the products and services they receive are useful, they are more likely to experience and immerse themselves in these products and services, and this sense of immersion helps to form a flow experience based on good perceived quality (Zhou et al., 2023; Joo et al., 2012). For example, in the process of online live streaming class learning, if users feel that the content of the classroom is very helpful to their learning and work, they are more likely to participate highly and experience flow.

When products and services are considered very useful, they are more likely to promote the occurrence of flow experience. This is because useful products and services can provide clear goals, potential control, immediate feedback, and the fusion of action and awareness, all of which are key elements of flow experience (Csikszentmihalyi, 1990). For example, if users believe that online live streaming class learning is very useful to them and the classroom quality is high, they are more likely to experience behaviors such as a sense of presence, time distortion, inattention, and loss of self-awareness, thereby improving learning effectiveness (Almaiah et al., 2022; Wang et al., 2021).

Furthermore, perceived usefulness also impacts the relationship between perceived quality and flow experience. If users believe that a product or service is useless to them, even if the quality is high, they are unlikely to experience flow. On the contrary, if users believe that the product or service is useful to them and the quality is also high, then the synergistic effect of perceived usefulness and perceived quality can promote the occurrence of flow experience (Jeong, 2011; Weibel et al., 2012; Rajagopal, 2022).

Therefore, we propose the following hypothesis:

H5: Perceived usefulness positively moderates the relationship between perceived quality and user flow experience during live online classroom participation.

**Figure 1:**
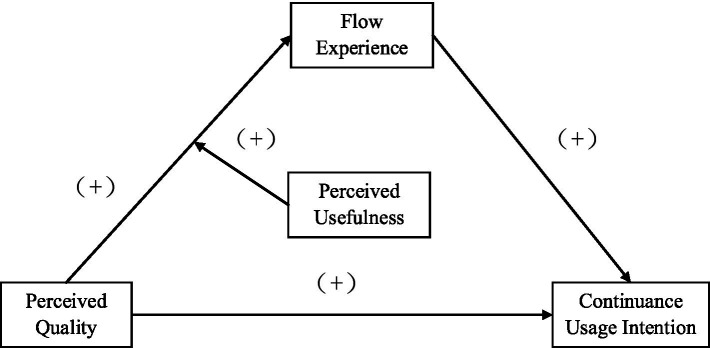
Theoretical model.

Based on the above analyses and the hypothesis from H1 to H5, we can initially draw the theoretical model of this article, as shown in Figure 1.

## 3 Methodology and data collection

### 3.1 Research design

This study focuses on the mechanism of the impact of perceived quality on users’ continuance usage intention in the context of online live streaming. Since the main variables involved in this article belong to the psychological perception level and are difficult to measure with public secondary data, we used the questionnaire survey method and the structural equation model based on the partial least squares method for research. Structural equation modeling (SEM) is a multivariate statistical analysis method used to test hypotheses about observed variables and latent variables. It can simultaneously implement multiple linear regression, principal component analysis and correlation analysis between two sets of variables. The structural equation model based on partial least squares (PLS) not only does not require the data to conform to the normal distribution, but also can handle complex structural models of multiple latent variables with both reflective indicators and formative indicators. Now it has been widely used in the fields of management information system, strategic management, organizational behavior, e-commerce and marketing.

To ensure that all questionnaire respondents have participated in online live classes, we carefully selected the target audience for the questionnaire distribution. In terms of questionnaire editing, we set questions around commonly used online live streaming class platforms to investigate user habits and demographic characteristics, and compared the results with industry survey reports. We collaborated with Tencent Questionnaire website (stock code: 0700.HK), leveraging its leading position in the internet field and rich user data to ensure that the characteristics of the questionnaire respondents match our research subjects. Before officially distributing the questionnaire, we commissioned Tencent Questionnaire website to conduct a small-scale pre-study to verify the data quality and the reliability of the questionnaire design. The results of the pre-study showed that the response rate and the quality of the questionnaire met expectations, and the questionnaire design could effectively screen out respondents who have truly participated in online live classes. Based on the results of the pre-study, we made minor adjustments to the questionnaire, and then carried out the formal questionnaire research and data collection. Through these measures, we ensured the reliability and validity of this study. We believe that by precisely selecting the target audience, cooperating with professional questionnaire services, and strictly verifying the pre-study, our data collection process can provide high-quality data support for the research.

### 3.2 Questionnaire development and pre-research

In this study, we mainly focused on potential variables including perceived quality, flow experience, perceived usefulness, and continuance usage intention, which also formed the main content of the questionnaire. Although these variables are difficult to measure directly, there are many well-established scales and literature from which to draw. Specifically, when setting the measurement items for continuance usage intention, we mainly drew on the research of Bhattacherjee (2001) and You et al. (2020). For the measurement of flow experience, we mainly draw on the research of Yeh et al. (2019), Jackson et al. (1999), and Chen (2006). For the measurement of perceived quality, we mainly draw on the literature of Dodds et al. (1991) and Agarwal and Teas (2002). Finally, when setting up the measurement items of perceived usefulness, we mainly drew on the papers of Gefen et al. (2003), Davis (1989), and Bhattacherjee (2001). After the preliminary development of the measurement scale was completed, we invited relevant members to form an expert group to design the measurement scale, including 4 postgraduate students, all of whom have experience using the online live class platforms, 1 doctoral student with experience in similar empirical research, and 1 industry expert with work experience in the platform experience department. The focus group discussed the measurement content of the initial scale, the wording of the items, the expression of the questions based on existing measurement questions, the conceptual interpretation of the research variables, and the scope that the indicators should cover. Based on the suggestions of the expert group, ambiguous items were modified. We also eliminated the questions that could not reflect the latent variable constructs, and ultimately formed the measurement scale of this paper. For all these questionnaire items, we used a 7-point Likert scale to measure the answers. Among them, 1 means strongly disagree and 7 means strongly agree. The specific questionnaire items and citations corresponding to each latent variable are shown in Table 1.

**Table 1:** Variables measurement and their references.

Latent variable	Code	Measurement	References
Perceived quality	PQ1	The fun of platform learning (virtual image, voice changer, funny expression pack, challenge to break through the barrier, etc.) exceeded my expectation.	Dodds et al. (1991), Agarwal and Teas (2002)
	PQ2	The functional experience of the platform (course recommendation, academic diagnosis, material management, etc.) is better than I expected.	
	PQ3	The courses on the platform meet my learning needs and exceed the expected results.	
	PQ4	The value-added services provided by the platform (professional assessment, micro-certificate, etc.) are very valuable, exceeding my expectations.	
	PQ5	Most of my expectations for the online live course platform have been met.	
Flow experience	FE1	I always know how the live online course is going.	Jackson et al. (1999), Chen (2006), Yeh et al. (2019)
	FE2	I know exactly what I should do during the live online class.	
	FE3	During the live online class, my attention was focused on the class.	
	FE4	Sometimes we ignore what is happening around us when we have a live online class.	
	FE5	During the live online class, I will be so absorbed that I forget that the time has passed by.	
	FE6	The time of the online live course passed much faster than I expected.	
	FE7	I will feel more happy in the online live course.	
	FE8	In the online live course, I found it quite interesting.	
Continuance usage intention	CUI1	I am willing to continue to use the online live course platform.	Bhattacherjee (2001), You et al. (2020)
	CUI2	I am willing to increase the frequency of using the online live course platform.	
	CUI3	I would like to continue to use the online live course platform without other alternatives.	
	CUI4	I have a positive evaluation of the online live course platform.	
	CUI5	I would like to recommend this online live course platform to others.	
Perceived usefulness	PU1	Online live streaming class can improve my learning efficiency.	Davis et al. (1989), Bhattacherjee (2001), Gefen and Straub (2003), Yan et al. (2021)
	PU2	Online live streaming class can improve my learning outcome.	
	PU3	Online live streaming class offers me the opportunity to learn more.	
	PU4	Overall, the online live streaming class are useful for my studies.	

In addition to the measurement items of the main variables mentioned above, the content of the questionnaire also contains two other parts. Among them, the first part is a survey on the use of online live broadcast classes, including the choice of specific online live streaming class platforms, frequency of use and reasons, to screen out respondents who frequently attend online live classes. The second part is the demographic information of the questionnaire fillers to observe and ensure that the respondents present a relatively even distribution.

We used the online questionnaire service of Tencent.com (stock code: 00700.hk) to conduct the survey. The initial study survey included individuals with a college degree or higher who were already employed. We entrusted Tencent.com sample service company to distribute questionnaires to target groups who have used the online live streaming class platforms before. During the 12-day survey period (October 4 to October 15, 2022), a total of 174 questionnaires were distributed, and 137 were returned, with a response rate of 79%.

Following the general procedure for screening questionnaire data, we checked the questionnaires and deleted those submitted within 2 min and those that appeared to be filled in at random. After screening, a total of 92 valid questionnaires that met the pre-screening criteria were obtained.

Through the analysis of the pre-survey data, we found that the four latent variables of continuance usage intention, perceived quality, flow experience and perceived usefulness can all be measured well. Overall, from the model adaptation results of confirmatory factor analysis, The Cronbach’s alpha coefficients (reliability tests) for the latent variables are all greater than 0.7, and the factor loadings corresponding to the observed indicators of each variable are all greater than 0.5. Therefore, the scales in this study have good reliability and validity and can be used in formal surveys.

### 3.3 Formal questionnaire research and data collection

Based on the results of the pre-survey, we conducted a formal survey using an adjusted questionnaire containing 22 measurement items. The distribution and collection of questionnaires were entrusted to the Tencent Questionnaire Platform from January 9 to January 15, 2023, during which a total of 1,107 responses were collected. Based on the data screening criteria (e.g., data defects, lack of live course experience, too short questionnaire response time), after excluding invalid data, a total of 773 valid questionnaires were collected, with an effective response rate of 70%. Table 2 describes the basic composition of the sample.

**Table 2:** The composition of the samples.

Variable	Category	Frequency	Percentage (100%)
Gender	Male	320	41
	Female	453	59
Age	Under 20	26	3
	20–25	416	54
	26–30	192	25
	31–40	84	11
	41–50	40	5
	Above 50	15	2
Major	Science and engineering	373	48
	Social sciences	200	26
	Liberal arts and history	200	26
Monthly income	Below 3,000 yuan	109	15
	3,000–6,000 yuan	397	51
	6,000–10,000 yuan	194	25
	Above 10,000 yuan	73	9

From Table 2 we can see that there are 320 male respondents and 453 female respondents, which indicates that our sample is well balanced in terms of gender ratio. In terms of the professional background of the questionnaire fillers, science and engineering majors and humanities and social science majors basically account for about 50% each, which can fully reflect the true situation of learning the content of various online live streaming classes. In addition, it can be observed that only 26 samples are under the age of 20, and most of the respondents are between the ages of 20 and 30. According to the user characteristic analysis in the CCTalk competitive analysis report, the age is mainly concentrated under 30 years old, which is the main group using live webcast courses. Moreover, from the perspective of income level, the income of most questionnaires is between 3,000 and 10,000 yuan, which is consistent with the resident income of ordinary working class released by the China Bureau of Statistics in 2023. Therefore, the formal survey data sample used in this study is well representative.

## 4 Results and discussion

### 4.1 Model measurement analysis

This article uses SPSS 24.0 and Smart PLS 4.0 to analyze the reliability and validity of the measurement model. First, we used SPSS 24.0 to conduct exploratory factor analysis (EFA). The KMO statistic is 0.977 and passed the test at the 99.9% significance level. The final co-precipitated factor is extracted using principal component analysis and the rotation method is Kaiser standardized maximum variance. Finally, 4 factors are extracted, with a cumulative contribution rate of 79.167% and factor loading are all above 0.6. Therefore, the indicators in this article have good convergent validity. In addition, the Cronbach’s *α* coefficients of all constructs are above 0.9 (see Table 3), ensuring the reliability of the scale in this study.

**Table 3:** Factor loading, CR and AVE of measurement model.

Latent variable	Measuring item	Factor loading	Cronbach’s *α*	C.R.	AVE
Perceived quality	PQ1	0.775	0.934	0.950	0.792
	PQ2	0.748			
	PQ3	0.778			
	PQ4	0.749			
	PQ5	0.801			
Flow experience	FW1	0.731	0.949	0.958	0.740
	FW2	0.740			
	FW3	0.754			
	FW4	0.770			
	FW5	0.693			
	FW6	0.746			
	FW7	0.755			
	FW8	0.753			
Continuance usage intention	CUI1	0.688	0.951	0.962	0.836
	CUI2	0.681			
	CUI3	0.638			
	CUI4	0.698			
	CUI5	0.702			
Perceived usefulness	PU1	0.704	0.930	0.951	0.829
	PU2	0.716			
	PU3	0.738			
	PU4	0.724			

Next, this study used Smart PLS 4.0 to conduct confirmatory factor analysis (CFA). The results showed that the goodness of fit of the model is acceptable (SRMR index value is 0.033, NFI is 0.954), and the factor loading is all above 0.60, both reaching a significance of 99.9% level. Besides, the average variance extracted (AVE) of all constructs is above 0.70, and the construct reliability (CR) is greater than 0.90, indicating that the constructs in this study have good convergent validity. In addition, as shown in Table 4, the square roots of all variables AVE are greater than the correlation coefficients between this construct and other constructs, indicating that the measurement model in this article has good discriminant validity (Discriminant Validity). Furthermore, this paper uses the Heterotrait-Monotrait ratio to evaluate discriminant validity, which is more sensitive to the validity of variance-based structural equations. It is found that the ratios are all lower than 0.85, which is lower than the recommended threshold (Henseler et al., 2015) (see Table 5). In short, the measurement model of this article meets the basic requirements of reliability and validity.

**Table 4:** Descriptive statistics, correlation analysis and validity test.

Variable	Mean	S.D	1	2	3	4
PQ	5.292	1.051	**0.890**			
FW	5.315	0.979	0.674**	**0.860**		
CUI	5.393	1.117	0.791**	0.786**	**0.915**	
PU	5.613	1.083	0.703**	0.776**	0.780**	**0.911**

***p < 0.001, **p < 0.01, *p < 0.05. The bold value at the diagonal is the AVE of the target variable.

**Table 5:** Heterotrait-Monotrait ratio.

Variable	CUI	FW	PQ	PU	PU × PQ
CI	–				
FW	0.827	–			
PQ	0.839	0.715	–		
PU	0.829	0.825	0.754	–	
PU × PQ	0.217	0.322	0.143	0.328	–

To examine the impact of multicollinearity, we checked the Variance Inflation Factor (VIF) values (Chin, 1998). The VIF values (inner VIF values) between the latent variables in this study are ranged from 1.129 to 2.195. Besides, the VIF values (outer VIF values) between indicators or measured items are ranged from 1.000 to 4.380. All these values are below the recommended threshold of 5.0, indicating that there is no serious multicollinearity issues (Hair et al., 2011).

### 4.2 Structural model analyses

**Figure 2:**
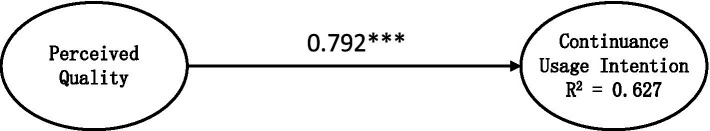
Perceived quality’s impact on continuance usage intention. ***p < 0.001.

The relationship between perceived quality and the continuance usage intention is the focus of this study. In order to test the hypothesis that perceived quality has a positive impact on the intention to continue to use, namely, hypothesis 1, we first constructed a structural equation model for only these two variables, and the empirical results are shown in Figure 2. The standardized root mean square residual (SRMR) measure of the model was 0.032 (< 0.05) and the Normalized Fit Index (NFI) value was 0.970 (> 0.90), indicating that the model fit well.

**Figure 3:**
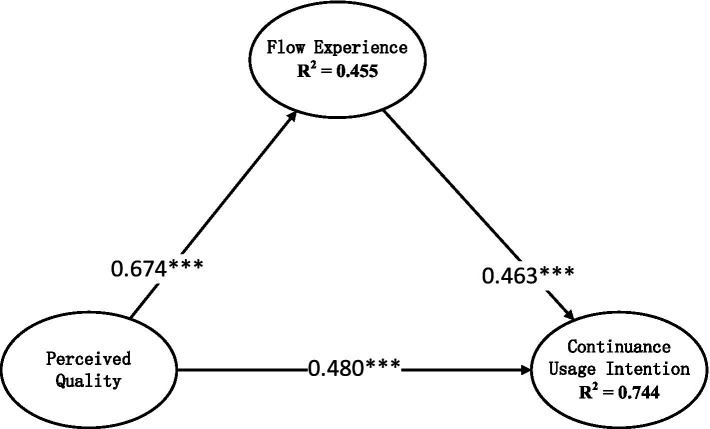
Perceived quality’s impact on continuance usage intention via flow experience. ***p < 0.001.

As can be observed from Figure 2, the standardized regression coefficient of perceptual quality to the user’s continuance usage intention (*β* = 0.792, *p* < 0.001) is significantly greater than 0, indicating that hypothesis 1 is validated. This shows that in the context of online live streaming classroom learning, perceived quality is an important factor for users to continue to use the platform. In order to better understand the mechanism of perceived quality on user sustained use, we introduced flow experience as a mediating variable and established a structural equation model, and the empirical results are shown in Figure 3. The SRMR measure of the model was 0.029 (< 0.05) and the NFI value was 0.961 (> 0.90), indicating that the model fit well.

As can be seen from Figure 3, compared with Figure 2, although the standardized regression coefficient of perceived quality on continuance usage intention has become smaller, but it is still significantly greater than 0. This may be due to the introduction of mediating variables. Therefore, let us observe the regression coefficient from the independent variable to the mediating variable. Obviously, the standardized regression coefficient of perceived quality on continuance usage intention is 0.674, which is significantly greater than 0. This indicates that hypothesis 2 has been verified. Next, let us take a look at the impact of flow experience on users’ willingness to continue using. Judging from the standardized regression coefficient, this value is also significantly greater than 0, indicating that flow experience has a significant positive impact on users’ continued usage intention. This shows that hypothesis 3 has also been confirmed. We can calculate the indirect effect of user perceived quality on users’ willingness to continue using the platform through flow experience as follows: 0.674 × 0.463 = 0.312. The 95% confidence interval is (0.033, 9.568) with the significance of *p* < 0.001, indicating the model’s indirect effect is significant. Combined with the direct effect of perceived quality on the user’s continuance usage intention of 0.480, we can calculate that the total effect of perceived quality on the user’s continuance usage intention is equal to 0.312 + 0.480 = 0.792. This is the total effect of perceived quality on the user’s continuance usage intention as shown in Figure 2’s regression coefficient.

The data analyses shown in Figures 2, 3 demonstrates the validity of Hypotheses 1, 2, 3 and 5. Moreover, it is not difficult to find that in the context of online live classroom learning, perceived quality is the most important factor for users’ continued use, explaining 62.7% of the variance change. In the Internet era that emphasizes user experience and service quality, creating a good perception of quality can directly and significantly increase customers’ willingness to continue using, which provides a clear direction for us to improve customer service levels. In the mediating path of perceived quality affecting users’ intention to continue using, we found that flow experience is a very important positive influencing factor. That is, in the context of online live classes, users’ quality perception can have an impact on their flow experience, and affect their continuance usage intention through flow experience, which has positive significance for how to improve users’ flow experience. In the field of service management and marketing, flow experience often has strong ambiguity and uncertainty. Most of the time, although it is known that flow experience has an important impact on user loyalty and repeat purchases, we still face many difficulties in how to improve flow experience. The research in this article shows that creating a good quality perception for online live classes can help improve users’ flow experience of teaching services. This reminds us that to improve users’ flow experience, we should not only start with the quality of the course itself, but also pay attention to the user’s perception of the quality of the course. On the other hand, the research in this article also proves the positive impact of flow experience on users’ continuance usage intention, which shows that in the context of online live streaming classes, flow experience is still an important factor in improving users’ continuance usage intention. While providers of online live streaming services should improve their own service levels, they should also pay more attention to the connection between service and user perceptions to improve users’ flow experience.

### 4.3 The moderating effect of perceived usefulness

**Figure 4:**
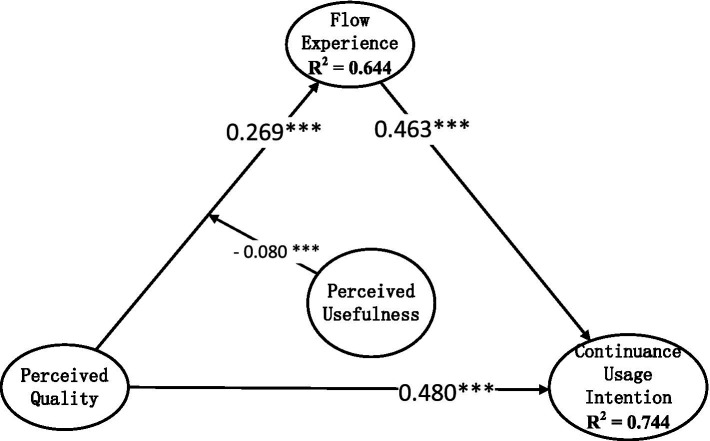
The moderating effects of perceived usefulness. ***p < 0.001.

The above-mentioned research shows that perceived quality can directly stimulate users’ continuance usage intention. In this process, it may be influenced by perceived usefulness. Perceived usefulness belongs to the perception of use value. Although all items with use value do not necessarily contain value, use value is still an important basis for value formation and may have an important impact on users’ flow experience. In order to verify the existence of the moderating effect of perceived usefulness, we added the moderating path of perceived usefulness on the relationship between perceived quality and flow experience based on the model in Figure 3, and continued to use the structural equation model based on partial least squares estimation method for analysis. The SRMR measure value of this model is 0.033 (less than 0.05), and the NFI value is 0.954 (greater than 0.90), indicating that the model fits well. The results are shown in Figure 4.

As can be seen from Figure 4, perceived usefulness has a significant negative moderating effect on the relationship between perceived quality and flow experience, and the standardized regression coefficient of the interaction term is −0.080. In other words, perceived quality can promote the formation of a good flow experience better in situations with weak perceived usefulness than in situations with strong perceived usefulness.

Improving users’ continuance usage intention through perceived quality requires consideration of specific situational factors. In this study, we proposed and validated the significant moderating role of perceived usefulness with empirical data. Previous literature has discovered many driving factors for the formation of flow experience, such as users’ personality characteristics, internal and external motivations, institutional support, etc. (da Silva deMatos et al., 2021; Khan et al., 2017; Demerouti et al., 2012). But a basic prerequisite for these factors to take full effect is that users believe that course learning will be useful to them. If users psychologically think that the course is not very useful to them, it will be difficult to participate deeply and develop a good flow experience. In response to this situation, our research found that the function of perceived quality began to emerge. If users have doubts and uncertainties about the usefulness of online live course learning, creating good platform functions and service quality for them can bring more improvements in flow experience than users who think course learning is useful. This finding provides an important new context for studying the value of perceived quality. In the context of online live streaming classroom learning, perceived quality can improve users’ streaming experience by creating a smooth and exciting usage experience for users of online live streaming classrooms. Especially when the user’s perceived usefulness is weak and it is difficult to touch the user’s heart at a high level, more investment should be made to provide users with better service quality to improve the user’s flow experience.

In fact, the influence between perceived quality and perceived usefulness is mutual. On the one hand, perceived quality can contribute to perceived usefulness. In turn, perceived usefulness can also improve the user’s perception of the quality of the live platform courses. On the other hand, good quality does not necessarily mean useful, and perceived quality does not necessarily lead to perceived usefulness. In the same way, what is useful is not necessarily of good quality, and the user’s perception of usefulness does not necessarily mean that the quality is also high. Regarding the negative moderating role of perceived usefulness on the relationship between perceived quality and flow experience, we considered that a fundamental prerequisite for the many factors affecting flow experience to play their roles is having perceived usefulness. If this prerequisite is met, it will enhance the impact of perceived quality on flow experience. Survey data show that the average score of perceived usefulness is 5.613, higher than the average score of perceived quality, which is 5.292. This indicates that the respondents generally have high perceived usefulness and perceived quality. However, why does the stronger perceived usefulness lead to a weaker impact of quality perception on flow experience? This is because, as we analyzed above, when users do not have a strong perceived usefulness to live streaming platforms and live classes, they will pay more attention to the flow experience brought by perceived quality. Therefore, in this case, the online live learning platform should pay more attention to and improve the perceived quality of users. Moreover, the emergence of flow experience does not only depend on perceived usefulness and perceived quality, but also depend on user personal characteristics, usage environment, task characteristics, and other factors.

## 5 Conclusion

In the Internet era, platform service quality and flow experience have become hot areas of academic research in recent years. During the coronavirus pandemic, online live streaming classes are increasingly becoming one of the main ways of teaching and learning at universities and further education and vocational training. Now, although the isolation control of the new coronavirus epidemic has been lifted, online live streaming classroom learning is still an important way for many students and professionals to supplement their knowledge and improve their skills. We commissioned a well-known Internet survey company to issue questionnaires and ensured the reliability of data collection. Data analysis results show that in the context of online live classroom learning, perceived quality has a positive impact on users’ continuance usage intention. During this process, flow experience plays an important mediating role. In addition, the effect of perceived quality on flow experience is also moderated by perceived usefulness. When users’ perceived usefulness is weak, the positive impact of perceived quality on continuance usage intention is stronger. These conclusions provide a useful reference for us to understand the mechanism of perceived quality in online live streaming classroom learning situations and the improvement of users’ continuance usage intention.

For online live streaming platforms, on the one hand, they should actively invest in improving the quality of user perception, as this can lead to a better user flow experience and continued willingness to use. This may be achieved by providing high-quality course content, optimizing user interface design, and ensuring smooth live streaming experiences. On the other hand, online live streaming platforms should also pay more attention to the user’s flow experience, as it also has a direct impact on the user’s propensity to continue to use. Platforms may design more engaging course content, offer personalized learning paths, and encourage interactions among users to facilitate the occurrence of flow experience. In addition, the online live streaming platform should also focus on the perceived usefulness of the online live class by the user. If users find live classes less useful, they should improve the quality of the courses. Because at this time, the quality of the course will have a greater effect on the user’s flow experience. Therefore, improving the quality of the course can increase the user’s intention to continue to use it more. Conversely, if users find live online courses useful, they should focus on improving other factors that affect users’ retention intentions while maintaining the quality of the course.

This study still has two major limitations. First, although this study confirmed the significant impact of perceived quality on continuance usage intention, it only explored the influencing mechanisms of flow experience and perceived usefulness. In fact, judging from the statistical results of this study, flow experience only mediates a small part of the total effect: 0.312/0.792 × 100% = 39.39%. This suggests that there may be other mediating variables. Considering the good application prospects of online live streaming scenarios in the future and consumers’ increasing attention to flow experience, we consider that it is necessary to further study whether there are other intermediary mechanisms between perceived quality and users’ continuance usage intention. Future research should also consider using more complex statistical models to explore the interaction between perceived usefulness, perceived quality, and flow experience, and consider including more variables that may affect flow experience, such as user personality traits, motivations, emotional states, etc. In addition, researchers should pay attention to the differences between different user groups and how these differences affect their evaluation of perceived usefulness and perceived quality of online live streaming classes. On the other hand, the empirical study in this article only uses survey data from one country. Although we commissioned a well-known questionnaire company, we cannot avoid the influence of cultural identities of different countries. Therefore, it is valuable to continue empirical research and exploration in countries with different cultural attributes and different levels of economic development.

## Data Availability

The raw data supporting the conclusions of this article will be made available by the authors, without undue reservation.
